# Wound Healing in Diabetic Patients Undergoing Abdominal Surgery: A Retrospective Study

**DOI:** 10.7759/cureus.90132

**Published:** 2025-08-15

**Authors:** Tiba Fadhil, Amna Batool, Hussain Khan, Muhammad Shahid Farooq

**Affiliations:** 1 General Surgery, Countess of Chester Hospital NHS Foundation Trust, Chester, GBR; 2 Surgery, Fatima Memorial Hospital, Lahore, PAK; 3 Molecular Medicine and Pathology, University of the Punjab, Lahore, PAK; 4 Pathology, Federal Postgraduate Medical Institute (PGMI), Lahore, PAK; 5 General Surgery, King Edward Medical University, Lahore, PAK

**Keywords:** abdominal surgery, diabetes mellitus, glycemic control, postoperative wound healing, surgical site infection (ssi)

## Abstract

Background

Patients with diabetes mellitus are more likely to develop complications in healing after abdominal surgery. The study aims to compare and evaluate the process of wound healing in diabetic and nondiabetic patients following abdominal surgery.

Methods

A group of 100 patients underwent elective abdominal surgeries (50 diabetic and 50 nondiabetic). A prospective observational study was performed on these patients. On days 3, 7, and 14, standardized wound healing scores were used to assess the process of wound healing after surgery. The CDC’s criteria were used to grade surgical site infections (SSIs) in this study. The factors observed for each sample were wound edema, erythema, discharge, the SSI grade, and the time it took to heal.

Results

Among the 100 patients who received abdominal surgery, the incidence of SSI was greater in diabetic patients, i.e., 15 (30%) versus five (10%) in nondiabetics. The healing time of >14 days was experienced in 20 (40%) diabetics compared to six (12%) nondiabetics, and dehiscence of wounds was found in six (12%) and two (2%), respectively (p < 0.05). The healing duration of diabetics was also longer (18.1 ± 4.6 vs. 12.5 ± 2.9 days). Grading of infection demonstrated greater Grade I: 10 (20%) vs. 4 (8%), Grade II: 4 (8%) vs. 1 (2%), and Grade III: 1 (2%) vs. 0 (0%). High BMI and diabetes were also major predictors of poor healing.

Conclusions

Diabetic people had worse postoperative wound healing after abdominal surgery. Glycemic control and perioperative strategies may help improve surgical outcomes.

## Introduction

The process of wound healing is a key determinant in surgical outcomes, especially in patients with long-term chronic systemic illnesses like diabetes [1]. Diabetes mellitus interferes with normal healing through mechanisms including persistent hyperglycemia, vascular dysfunction, impaired immune responses, and defective leukocyte function [2,3]. These factors lead to slow wound healing, a higher likelihood of surgical site infections (SSIs), and longer hospital stays. Large cuts made during abdominal surgery and extra handling of tissues can cause more problems at the wound site in diabetic people. As the prevalence of diabetes continues to increase around the world, there is an increased demand to comprehend the challenges of wound healing that are unique to diabetic patients [4,5].

Inadequate blood sugar management after surgery leads to increased infections, affects collagen production, and inhibits the growth of blood vessels, all of which negatively influence the recovery of tissues [6,7]. Having surgery when you have diabetes and inflammation makes wound healing more complex, so clinicians should pay extra care and manage these patients properly. Regardless of new surgical and wound management practices, people with diabetes tend to have more problems after surgery compared to those without diabetes [8]. Despite the established pathophysiology of poor wound healing in diabetes, there is a lack of evidence on the role of perioperative glycemic control, specifically on wound healing outcomes and severity of infection following abdominal surgery.

Therefore, this study evaluates wound healing in diabetic patients after abdominal surgery, including the rate of wound healing and the association with the patients’ ability to control their infection. Early detection of infection and subsequent actions can make a significant difference in the healing and all aspects of care.

## Materials and methods

This retrospective observational study by a tertiary care hospital (September 2023 to December 2023) analyzed wound healing among diabetic patients who underwent abdominal surgery. A total of 100 participants, including diabetic patients, were enrolled through a consecutive sampling method. The sample size was calculated by OpenEpi 3.0.0 (Released 2013, Atlanta, GA, USA) with an estimated diabetes infection rate of 20%, 95% CI, and 80% power. The research study was ethically approved by the affiliated hospital, School of Allied Health Sciences, University of the Punjab, Lahore (approval 1449-23).

Inclusion criteria were type 1 or type 2 diabetes mellitus, age between 30 and 75 years, and elective abdominal surgery or emergency abdominal surgery (minor) with informed consent. Exclusion criteria were immunosuppressive medications, having a medical history of malignancy, having any autoimmune diseases, or having incomplete documentation for the scope of this study. To detect glycemic control, the preoperative measurement of baseline fasting and random blood glucose levels was determined. Senior surgical staff assessed postoperative outcomes through surgical wounds that were examined on a daily basis. The severity of SSIs via the Clinical Severity Scale was also graded on an established scale: Grade 0, there was no sign of infection; Grade 1, there was mild erythema or serous discharge; Grade 2, there was an infection that required an antibiotic; and Grade 3, this was an infection that required drainage or surgical treatment. The wound healing duration was expressed as the cumulative days of observation to attain full epithelialization and the absence of any form of discharge. The data gathered were age, sex, type and duration of surgery, preoperative glycemic status, SSI grade, and total healing time, which are clinical variables. All the surgical activities and postoperative assessments were carried out by trained personnel according to standard protocols.

The analysis of all data was performed with the help of IBM SPSS Statistics for Windows, Version 26.0 (Released 2018; IBM Corp., Armonk, NY, USA). Demographic and clinical characteristics were described using descriptive statistics (mean, SD, frequencies, and percentages). Associations between categorical variables were examined with the chi-square test, including glycemic control and the occurrence of SSI. An independent t-test or ANOVA was used to compare continuous variables where necessary. The statistical significance was set at a p-value < 0.05.

## Results

For this study, 100 patients who underwent abdominal surgeries were examined: 50 diabetics and 50 nondiabetics. Standard clinical signs were used to evaluate postoperative wound healing on days 3, 7, and 14. At the beginning of the analysis, it was evident that both groups experienced different rates of wound healing, infection, and recovery.

Table 1 demonstrates the baseline demographic and clinical characteristics.

**Table 1:** Patient clinical baseline characteristics (n = 100)

Variable	Diabetic (n = 50) (mean ± SD)	Nondiabetic (n = 50) (mean ± SD)	Test used	Test value	p-Value
Mean age (years)	59.2 ± 8.1	56.8 ± 7.9	t-test	t = 1.45	p = 0.15
Male (%)	30 (60%)	29 (58%)	Chi-square test	χ² = 0.04	p = 0.84
BMI (kg/m²)	28.7 ± 3.4	26.5 ± 3.1	t-test	t = 3.55	p = <0.001
Hypertension (%)	34 (68%)	21 (42%)	Chi-square test	χ² = 7.2	p = 0.007

The diabetic group had a slightly higher mean age (59.2 ± 8.1 years) compared to the nondiabetic group (56.87 ± 7.9 years), with no significant difference. The groups had similar gender distributions. Nevertheless, BMI (28.7 ± 3.4 kg/m² and 26.5 ± 3.1 kg/m², p = 0.0006) and hypertension (34 (68%) and 21 (42%), p = 0.007) were higher in diabetic patients, and therefore, diabetics had a higher burden of comorbidities.

The distribution of SSI grades across the study participants is presented in Table 2.

**Table 2:** SSI grades SSI, surgical site infection

SSI grade (CDC classification)	Diabetic (n = 50), N (%)	Nondiabetic (n = 50), N (%)	Test used	Test value	p-Value
Grade I (superficial)	10 (20%)	4 (8%)	Chi-square test	χ² = 3.27	p = 0.07
Grade II (deep incisional)	4 (8%)	1 (2%)	Chi-square test	χ² = 1.39	p = 0.23
Grade III (organ/space)	1 (2%)	0 (0%)	Fisher’s exact test	-	p = 0.31
No infection	35 (70%)	45 (90%)	Chi-square test	χ² = 6.25	p = 0.012

There was a higher rate of superficial infections (Grade I) in diabetic patients, which were present in 10 (20%) of the diabetic patients but in four (8%) of the nondiabetic patients. Infections deep into the incisions (Grade II) were also more common in diabetic cases, with four (8%) cases as compared to one (2%) nondiabetic case. Moreover, one (2%) diabetic patient also had organ/space infections (Grade III), whereas no cases were found in nondiabetics. The likelihood of the percentage of patients without an infection was significantly lower in diabetics (35; 70%) than in nondiabetics (45; 90%), which implies that diabetic patients had a burden of infections.

The overall wound healing outcomes are shown in Table 3.

**Table 3:** Wound healing outcomes SSI, surgical site infection

Outcome	Diabetic group	Nondiabetic group	Test used	Test value	p-Value
SSI	15 (30%)	5 (10%)	Chi-square test	χ² = 6.25	p = 0.012
Delayed healing (>14 days)	20 (40%)	6 (12%)	Chi-square test	χ² = 10.76	p = 0.001
Wound dehiscence	6 (12%)	1 (2%)	Chi-square test	χ² = 4.08	p = 0.043
Mean healing time (days)	18.1 ± 4.6	12.5 ± 2.9	t-test	t = 7.36	p = <0.001

The incidence of SSIs was much higher in diabetic patients (15; 30%) than in the nondiabetic group (5; 10%). Delayed healing, taking more than 14 days to recover, was observed in 20 (40%) diabetic patients and six (12%) nondiabetic patients. The wound dehiscence was observed in six (12%) and one (2%) patients in the diabetic and nondiabetic groups, respectively, and the difference between the groups was found to be significant. There was also a significant increase in the mean healing time in diabetics (18.1 ± 4.6 days) compared to nondiabetics (12.5 ± 2.9 days), implying that there was a substantial delay in the healing process among the diabetics.

The risk factors for poor healing are described in detail in Table 4.

**Table 4:** Key risk factors for poor healing

Factor	Associated with poor healing?
Diabetes	Yes
High BMI	Yes
Smoking	No
Hypertension	No

Diabetes and high BMI were among the significant predictors of slow healing after abdominal surgery. Conversely, smoking and high blood pressure did not show a significant relationship with poor healing outcomes in this study population. These results suggest the significance of metabolic factors, especially obesity and glycemic control, in predicting surgical recovery.

## Discussion

The aim of this study was also to assess the effect of diabetes mellitus on wound healing outcomes after abdominal surgery. Findings of this study indicated that diabetic patients were more likely to get SSIs, slow healing, and wound complications compared to nondiabetic patients. These results indicate that microvascular dysfunction, impaired immune system, and chronic inflammation, often maintained in diabetes, are key factors in worsening the process of recovery after surgery [9]. The results of this research align with more stable physiological processes, including detrimental consequences of hyperglycemia in leukocyte response, collagen production, and neovascularization [10].

The current study is in line with the recent findings that demonstrated an increase in wound complications among diabetic surgical patients [11]. According to previous studies, the prevalence of SSIs was much higher in patients with diabetes who received abdominal surgery, which is consistent with our data [12]. Likewise, a study found that diabetic patients with complex abdominal operations have more wound-related morbidity and prolonged recovery periods [13]. Both studies validate the belief that diabetes, especially in poorly controlled cases, is one of the factors in poor surgical outcomes [14]. Additionally, the contribution of diabetes to abnormalities of the wound microenvironment, including augmented reactive oxygen species and advanced glycation end-products and sustained expression of inflammatory cytokines, interferes with fibroblast activity and re-epithelialization, impairs wound closure, and predisposes to wound infection [15,16]. There is additional clinical evidence that intervention therapies, such as negative pressure wound therapy or application of growth factors and skin substitutes, can be applied as adjunctive modalities to increase healing rates in diabetic patients following abdominal surgery [17]. Such emerging tactics raise the possibility of more personalized surgical care among patients with diabetes.

This study, however, did not show any significant relationship between hypertension, smoking, and delayed wound healing, as some of the recent investigations indicate a contributory role of the vascular comorbidities [18]. This discrepancy may be attributed to the sample size or the exclusion criteria used. Importantly, BMI was identified as a key predisposing element to poor healing, which agrees with recent studies indicating that obesity is associated with poor perfusion and tissue oxygenation [19,20].

One of the major limitations of this study was the lack of stratified data on glycemic control (e.g., HbA1c grouping), which restricts the possibility of examining the direct association between glycemic control and healing. Moreover, it was a single-center investigative study with a small sample size and the follow-up duration of only 14 days. The influence of other confounders, including surgical technique, nutritional status, and length of surgery, which are known to impact wound healing, was not analyzed. Future investigations need larger multicenter studies with longer follow-ups and necessary classification according to glycemic parameters.

## Conclusions

Current research observed a higher incidence of SSI, slow wound healing, and wound dehiscence in diabetic patients undergoing abdominal surgery compared to nondiabetic patients. The study outcomes emphasized the substantial impact of diabetes on postoperative wound and tissue healing. The observed results of differences in wound complications between diabetic and nondiabetic patients support the interpretation that diabetes mellitus may be a significant indicator of the delayed postoperative wound recovery following abdominal surgery.
